# Supplementary material for: The 1 –Cys peroxiredoxin, PRDX-6, suppresses an NHR-49-dependent pro-survival response, including the Flavin monooxygenase, FMO-2, that protects against fungal and bacterial infection
Source: Redox Biol. 2025 Dec 29;91:103992. doi: 10.1016/j.redox.2025.103992 (PMC12930036; doi:10.1016/j.redox.2025.103992)
Supplement: Multimedia component 1 [file mmc1.pdf]

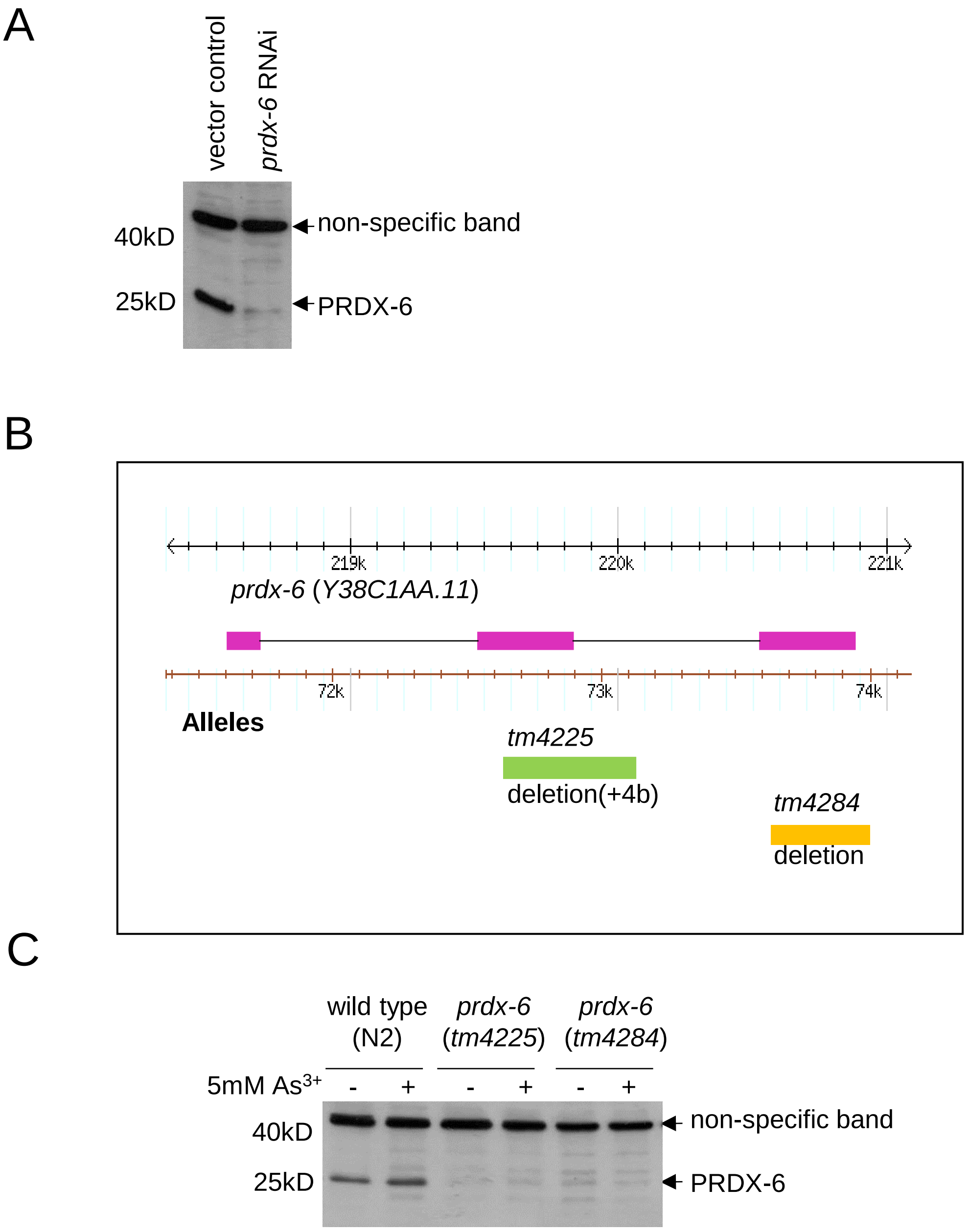

Fig. S1 Antibody 5754-1-4M234/4J10_120208 detects a protein predicted to be PRDX-6 that is reduced by *prdx-6* RNAi and absent from *prdx-6* mutant worms. [A] Immunoblot of wild type worms treated with vector control and *prdx-6* RNAi with anti-PRDX-6 antibodies indicates that *prdx-6* RNAi reduces the levels of a protein of the expected size(25.6kDa). [B] The genome structure of the *prdx-6* gene indicating the regions that are deleted in worms bearing the *tm4225* and *tm4284* alleles [C] Western blot of protein lysates of wild type, (N2) *prdx-6 (tm4225)* and *prdx-6 (tm4284)* mutant worms before and after treatment with 5mM arsenite for 5 minutes and probed with antibody 5754-1-4M234/4J10_120208. The absence of the 25kDa band from lysates from *prdx-6 tm4225* and *tm4284* mutants confirming loss of the PRDX-6 protein. A non-specific band around 40kD is also indicated, confirming even protein loading.

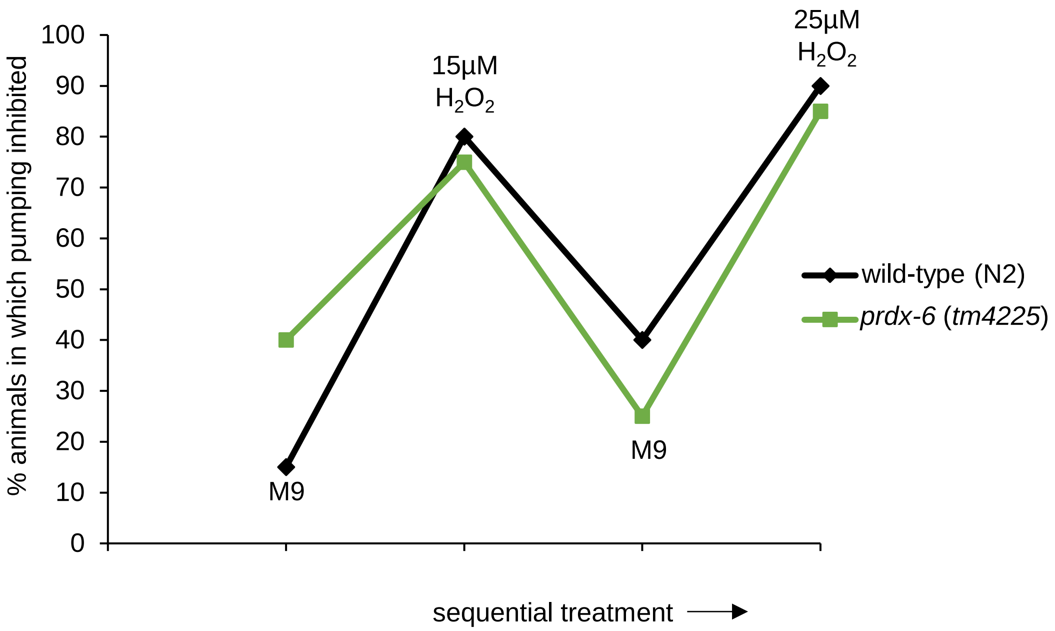

Fig. S2 PRDX-6 is not required for inhibition of pumping in response to H_2O_2. When young adult animals were sequentially exposed to a droplet of M9 (control), 15µM H_2O_2, M9 then 25µM H_2O_2 a similar % of wild-type and *prdx-6* (*tm4225*) mutant animals exhibited transient inhibition of pumping in response to H_2O_2 (n=20 in each group). Pumping inhibition was assayed essentially as previously described (Bhatla and Horvitz, 2015): A droplet of M9 or M9 containing hydrogen peroxide was added close to the animal, using a pipette or a needle, such that the liquid engulfed the head only. The response was scored by eye on a stereoscope and inhibition was determined upon a noticeable pause in the rhythmic pumping of the pharyngeal grinder within 10s of liquid entering the pharynx. Experiment was repeated with similar results and a representative experiment is shown.

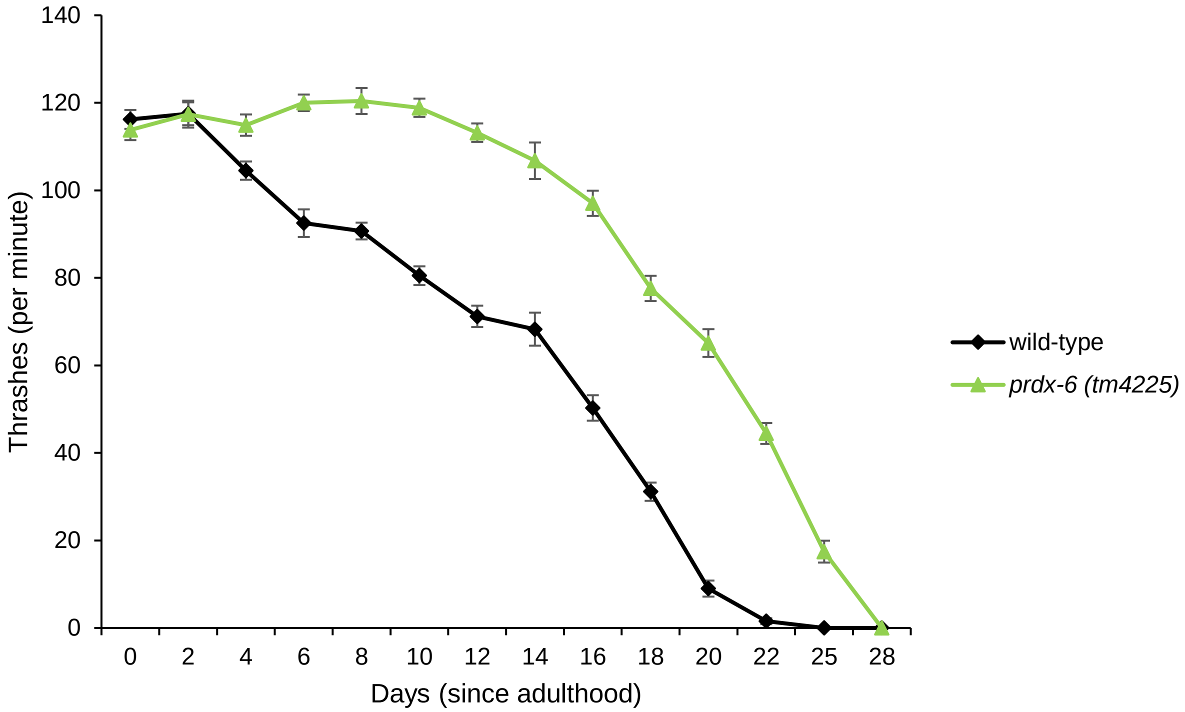

Fig. S3 PRDX-6 mutant animals exhibit a slower decline in mobility than wild-type animals. The motility of 30 young adult wild-type and 30 *prdx-6 (tm4225)* animals maintained at 15°C was assessed daily by counting thrashes over 20s following immersion in 3µl M9, as described in Leiser et al 2015. Mean thrashes per minute were calculated. Error bars represent the standard error of the mean.

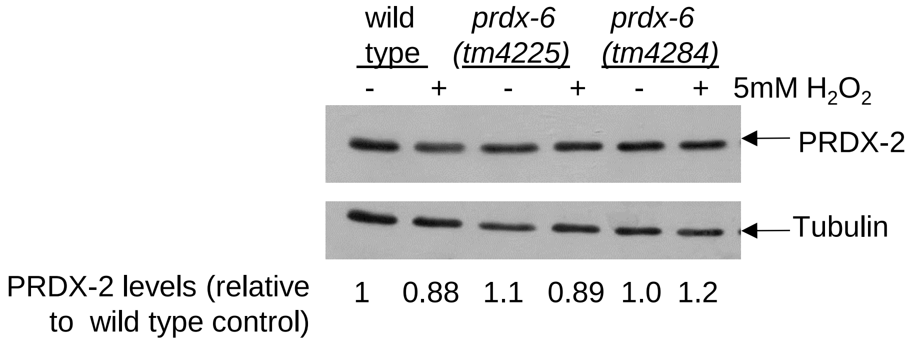

Fig. S4 Wild type and *prdx-6* mutant worms contain similar levels of PRDX-2. Western blot analysis of wildtype, *prdx-6* (*tm4225*) and *prdx-6* (*tm4284*) mutant worms before and after 5 min exposure to 5 mM H_2O_2 with anti-PRDX-2 antibody. The tubulin antibody was used as a loading control to normalise PRDX-2 levels using ImageJ software. The average quantified levels of PRDX-2 compared to the unstressed wild type from two repeats are displayed beneath the Western Blot. T-test revealed that none of the groups were significantly different to wild type unstressed, $p<0.05$

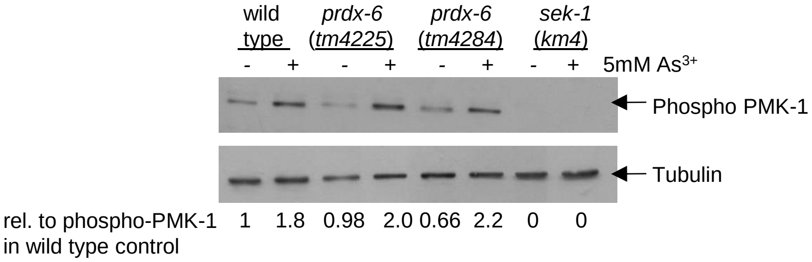

Fig. S5 Basal and arsenite induced increases in PMK-1 phosphorylation were not significantly affected by loss of *prdx-6*. Western blot of wildtype (N2), *prdx-6* (*tm4225*) and *prdx-6* (*tm4284*) mutant worms before and after 5 minutes exposure to 5mM As^{3+} probed with an antibody specific to phosphorylated PMK-1 as confirmed by the absence of a band in *sek-1* (*km4*) mutant animals. The tubulin antibody was used as a loading control to normalise PMK-1 phosphorylation using ImageJ software. The average quantified levels of PMK-1 phosphorylation compared to the unstressed wild type from three repeats are displayed beneath the Western Blot. T-test revealed that none of the groups were significantly different to wild type, $p<0.05$

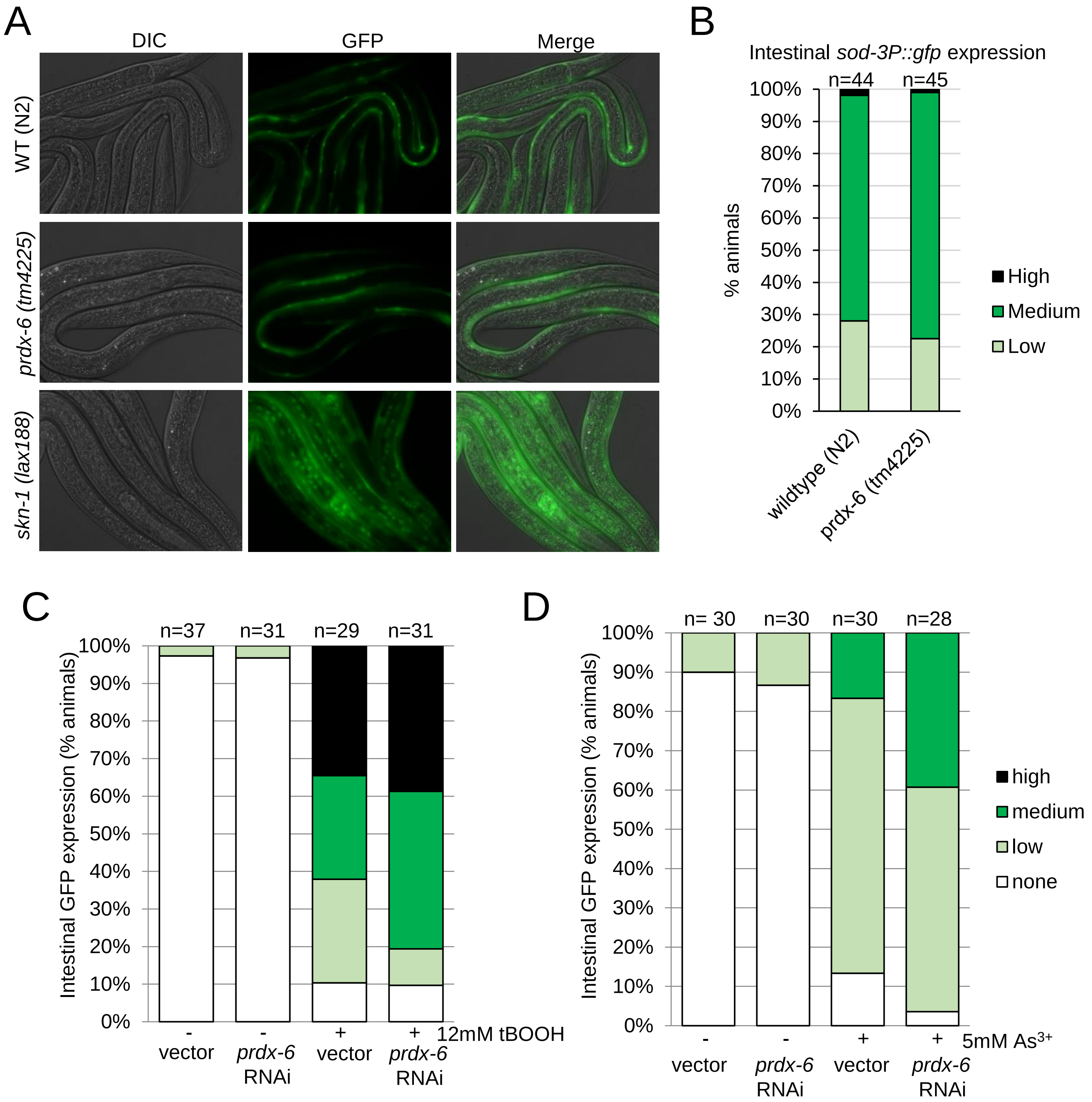

Fig. S6 Loss of *prdx-6* does not affect expression of SKN-1 and DAF-16-activated transcriptional reporters. [A] Analysis of *gst-4P::*GFP expression in wild-type and *prdx-6* mutant larval animals compared with animals expressing a skn-1 gain of function allele. **[B]** The intestinal expression of *sod-3P::*GFP was scored in wild-type and *prdx-6(tm4225)* mutant L3 animals. **[C-D]** The intestinal expression of *gcs-1*P::GFP was scored in L4 wild type animals treated with either vector control or *prdx-6* RNAi, without stress and stressed with [C] 12mM tBOOH or [D] 5mM arsenite (As^{3+}) The experiment was performed twice with similar results and a representative experiment is shown. Chi-squared test compared vector control treated worms to *prdx-6* RNAi treated, [C] without stress p=0.90; with tBOOH stress p=0.31 [D] without stress p=0.69; with arsenite stress p=0.097 n= number of animals per group.

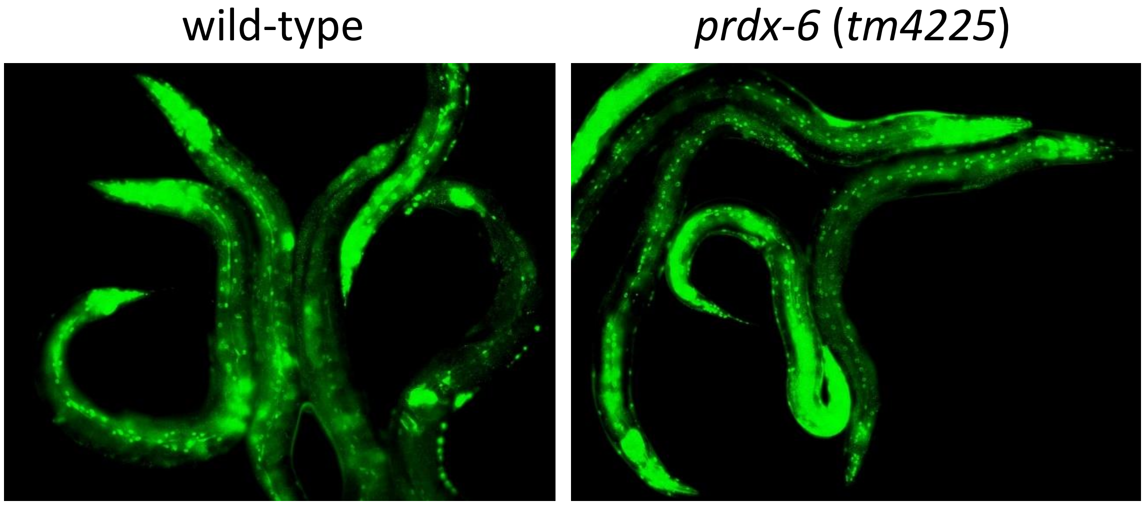

Fig. S7 NHR-49+GFP levels and distribution are similar in *wild type and prdx-6 mutant C. elegans* NHR-49::GFP expression was observed in well-fed wild-type (AGP24) and *prdx-6* (*tm4225*) mutant animals expressing *Pnhr-49::nhr-49::gfp* (Ratnappan et al. 2014). More than 100 adult animals were examined and imaged under identical conditions/exposures. Although there was some variation between animals in each group, no differences were observed.. Representative animals from each group are shown.

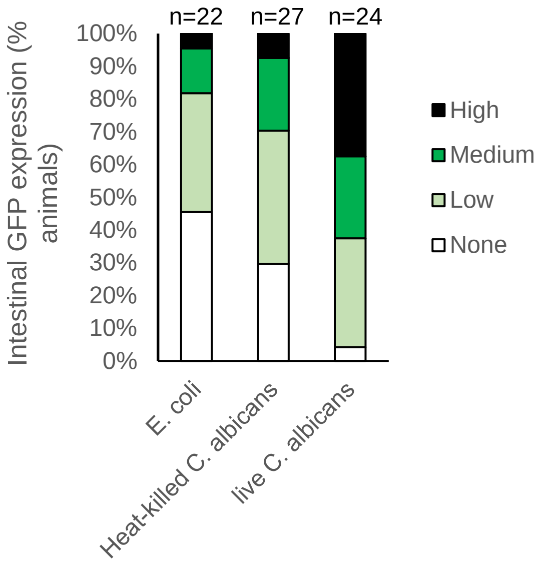

Fig. S8 *fmo-2P::gfp* expression in wild type worms on *E. coli* or following 6h exposure to live or heat-killed *C. albicans.* The intestinal expression of *fmo-2P*::GFP was scored in L4 wild type animals maintained on *E. coli* (OP50) 6hour following transfer to *E.coli,* live or heat-killed *C. albicans.* Chi^2 test comparing expression in animals treated with *E. coli* or heat-killed *C. albicans* p=0.674 whereas exposure to live *C. albicans* significantly increased intestinal *fmo-2P*::GFP expression compared with *E. coli* (chi^2 test p=0.0021) or heat-killed *C. albicans* (chi^2 test p=0.017). n=number of animals per group.

Table S1 Lifespan parameters associated with survival monitoring experiment shown in Fig. 4C (lifespan at 15°C)

**Strain**	**Mean** (days)	**Median** (days)	**Log Rank p-value (relative to wildtype)**
wild type (N2)	24.12	24	
prdx-6 (*tm4225*)	30.63	32	<0.001
prdx-6 (*tm4284*)	33.45	32	<0.001

Table S2 Lifespan parameters associated with survival monitoring experiment shown in Fig. 4D (lifespan at 25°C)

**Strain**	**Mean** (days)	**Median** (days)	**Log Rank p-value (relative to wild type)**
wild type (N2)	6.69	6	
prdx-6 (*tm4225*)	7.34	7	0.447
prdx-6 (*tm4284*)	7.61	7	0.132

Table S3 Combined lifespan parameters from 3 independent lifespan experiments at 15°C and 25°C

	**15°C**			**25°C**		
**Strain**	Mean Lifespan (days)	Median Livespan (days)	no. of worms	Mean Lifespan (days)	Median Livespan (days)	No. of worms
N2	24.7	24	187	7.26	7	112
prdx-6 (*tm4225*)	29.1	30	159	7.50	7	115
prdx-6 (*tm4284*)	29.5	30	156	7.40	7	108

Table S4 Loss of PRDX-6 causes an increased sensitivity to *S*. Typhimurium (parameters related to experiment shown in Fig. 5A)

**Strain**	**no. of worms**	**Mean survival** (days)	**Log Rank p-value (relative to wild type)**
wild type (N2)	75	6.87	
prdx-6 (*tm4225*)	57	5.07	<0.001
prdx-6 (*tm4284*)	69	5.17	0.001

Table S5 Loss of PRDX-6 causes an increased resistance to *S. aureus* (parameters related to experiment shown in Fig. 5B)

**Strain**	**no. of worms**	**Mean survival** (days)	**Log Rank p-value (relative to wild type)**
wild type (N2)	70	1.77	
prdx-6 (*tm4225*)	66	3.47	<0.001
prdx-6 (*tm4284*)	63	3.03	<0.001

Table S6 Loss of PRDX-6 causes an increased resistance to *C. albicans* (parameters related to experiment shown in Fig. 5C)

**Strain**	**no. of worms**	**Mean survival** (days)	**Log Rank p-value (relative to wild type)**
wild type (N2)	109	2.55	
prdx-6 (*tm4225*)	125	3.86	<0.001
prdx-6 (*tm4284*)	112	3.55	<0.001

Table S7 Effect of loss of PRDX-6 on the hypersensitivity of *pmk-1* mutants to *S. aureus* infection (parameters related to experiment shown in Fig. 5E)

**Strain**	**no. of worms**	**Mean survival** (days)	**Log Rank**	**p-value**
			cf wt	cf *pmk-1*
wild type (N2)	59	2.55		
prdx-6 (*tm4225*)	31	3.86	<0.001	
prdx-6 (*tm4284*)	28	3.55	<0.001	
pmk-1 (km25)	52	1.26	<0.001	
prdx-6 (*tm4225*) *pmk-1 (km25)*	25	1.32		0.637
prdx-6 (*tm4284*) *pmk-1 (km25)*	45	1.46		0.063

Table S8 Effect of loss of PRDX-6 on the hypersensitivity of *pmk-1* mutants to *C. albicans* infection (parameters related to experiment shown in Fig. 5F)

**Strain**	**no. of worms**	**Mean survival** (days)	**Log Rank**	**p-value**
			cf wt	cf *pmk-1*
wild type (N2)	107	1.93		
prdx-6 (*tm4225*)	90	4.40	<0.001	
prdx-6 (*tm4284*)	97	3.31	<0.001	
pmk-1 (km25)	102	1.24	<0.001	
prdx-6 (*tm4225*) *pmk-1 (km25)*	103	2.15		<0.001
prdx-6 (*tm4284*) *pmk-1 (km25)*	103	1.87		<0.001

Table S9 FMO-2 increases resistance to *S. aureus* (parameters related to experiment shown in Fig. 6D)

**Strain**	**no. of worms**	**Mean survival** (days)	**Log Rank p-value (relative to wild type)**
wild type (N2)	113	2.86	
fmo-2 (*ok2147*)	112	2.22	<0.001
fmo-2oEx	116	3.22	<0.001

Table S10 FMO-2 increases resistance to *C. albicans* (parameters related to experiment shown in Fig. 6E)

**Strain**	**no. of worms**	**Mean survival** (days)	**Log Rank p-value (relative to wild type)**
wild type (N2)	98	2.32	
fmo-2 (*ok2147*)	89	1.91	=0.008
fmo-2oEx	92	4.09	<0.001

Table S11 Lifespan parameters associated with survival monitoring experiment shown in Fig. 7D (lifespan at 15°C)

**Strain**	**no. of worms**	**Mean survival** (days)	**Median survival** (days)	**Log Rank p-value (relative to wildtype)**
wild type (N2)	56	22.71	23	
prdx-6 (*tm4225*)	53	26.94	27	<0.001
fmo-2 (ok2147)	59	26.75	27	<0.001
prdx-6 (tm4225)<br>*fmo-2 (ok2147)*	53	28.1	30	<0.001

Table S12 FMO-2 is not required for increased resistance to *S. aureus* in *prdx-6 (tm4225)* animals (parameters related to experiment shown in Fig. 7E)

**Strain**	**no. of worms**	**Mean survival** (days)	**Log Rank p-value (relative to wild type)**
wild type (N2)	36	6.42	
prdx-6 (*tm4225*)	32	8.5	<0.001
fmo-2 (ok2147)	36	5.78	=0.751
prdx-6 (tm4225) fmo-2 (ok2147)	30	8.2	<0.001
